# Commentary on the T1D exchange quality improvement collaborative learning session November 2022 abstracts

**DOI:** 10.1111/1753-0407.13327

**Published:** 2022-11-02

**Authors:** Shivani Agarwal, Nicole Rioles, Shideh Majidi, Robert Rapaport, Osagie Ebekozien, for the T1DX‐QI Collaborative

**Affiliations:** ^1^ Fleischer Institute for Diabetes and Metabolism Albert Einstein College of Medicine, Montefiore Medical Center Bronx New York USA; ^2^ NY‐Regional Center for Diabetes Translational Research Albert Einstein College of Medicine, Montefiore Medical Center Bronx New York USA; ^3^ T1D Exchange Boston Massachusetts USA; ^4^ Children's National Hospital Washington District of Columbia USA; ^5^ Mount Sinai Kravis Children's Hospital Icahn School of Medicine New York New York USA; ^6^ University of Mississippi School of Population Health Jackson Mississippi USA

**Keywords:** health equity, learning health systems, quality improvement, type 1 diabetes

## 1 INTRODUCTION

The T1D Exchange Quality Improvement Collaborative (T1DX‐QI) continues to be a leader in driving innovation advancements in type 1 diabetes (T1D) care.^1^ With over 50 diabetes centers in its consortium across the United States, including 32 pediatric and 18 adult centers, T1DX‐QI has been able to capture T1D electronic medical record data on over 55 000 people with T1D. Through patient and parent partners, an engaged group of multidisciplinary healthcare providers, and an advisory board specifically focused on racial‐ethnic equity, T1DX‐QI has been able to glean vital insights about real‐world diabetes care and outcomes, especially among those underserved and traditionally excluded from research.^2^

This year′s quality improvement conference covers some of the most pressing issues in diabetes care, with a focus on using technology to improve outcomes in high‐risk underserved populations.

Several abstracts, contributed by Noland, Lockee, Kaplin, and Izquierdo, describe use of big data and artificial intelligence algorithms to identify patients at high risk for a variety of complications, including poor glycemic outcomes, hospitalizations for diabetic ketoacidosis, and long‐term complications.^3^, ^4^, ^5^, ^6^ One abstract by Vandervelden details how the development of a T1D dashboard enabled better population health management through identification and systematic tracking of high‐risk patients.^7^ A new QI portal dedicated to improving healthcare clinic self‐monitoring and facilitating sharing of ideas has been described. This may be a model for other learning health networks to build cross‐collaboration.^8^ These ways of harnessing real‐world data have the potential to identify new at‐risk populations and drive change in clinical care using QI methodologies.^9^, ^10^

Another theme of the abstracts has been a hot topic in much of the literature: continuous glucose monitoring and insulin pump equity in T1D were explored by Adams, Gandhi, Wong, and Mathias.^11^, ^12^, ^13^, ^14^ Inequity in diabetes technology has been demonstrated in numerous studies from the T1D Exchange network based on the US population^15^ and in comparison to a population from the German/Austrian Diabetes‐Patienten‐Verlaufsdokumentation registry.^16^ These and other papers underscore how critical and pervasive the inequity remains. Although acknowledgement of these issues continues to be vitally important, what is exciting about the abstracts this year is that many diabetes centers have started to develop and test solutions to improve technology use among underserved populations.^11^, ^12^, ^13^, ^14^ Interventions ranging from better outreach and tracking of patients who are eligible but may not be using technology^16^ to race‐targeted approaches that change workflows or lower barriers for technology eligibility^17^, ^18^ and modification of clinical pathways to prescribe, authorize, and support underserved patients while using technology are all explored by Wong, Virani, and Byer‐Mendoza.^13^, ^19^, ^20^ Future work will continue to enable cross‐pollination of interventions such that a suite of possible solutions to form a change package may eventually be developed to be employed by clinics outside of the network.

The last major focus of abstracts is the important topic of screening for social determinants of health in diabetes care. Multiple clinics have explored how to incorporate social determinants of health screening into current workflows,^10^, ^11^, ^19^, ^21^, ^22^, ^23^ some using manual processes, others using electronic medical record systems and leveraging institution‐wide initiatives in primary care or pediatrics to achieve specialty‐care level reach.^10^ Overall, these abstracts show that screening for social determinants of health is feasible; valued by providers, patients, and institutions; and has potential for significant impact on addressing the unmet needs of vulnerable populations who are often at highest risk for short‐ and long‐term complications.

## 2 SUMMARY

This year has been an exciting time of major strides for the T1D Exchange Collaborative, keeping current issues at the forefront of clinical real‐world care. T1DX will continue to search for new ways to drive change for patients and their families, using novel QI methodology, stakeholder engagement, and data‐driven approaches. Future directions may include diabetes technology data integration in the electronic medical record, new psychosocial care models that address highly prevalent psychological issues in people with T1D, and shared decision aids that promote patient‐centered care in diabetes.

## AUTHOR CONTRIBUTIONS

OE conceptualized the manuscript. SA wrote the manuscript. SM, NR, and RR reviewed/edited and approved final versions of the manuscript. OE, NR are the guarantors of this work.

## DISCLOSURE

SA is a healthcare disparities advisor for Beta Bionics and Medtronic. NR has no disclosures. SM has no disclosures. OE is a member of the Medtronic Diabetes Health Equity Advisory Board; He is the Principal Investigator for research projects funded by Eli Lilly &Co, Medtronic Diabetes, Abbott Laboratories, and Dexcom Inc. All the funds for these industry‐funded projects and board roles are paid directly through his organization, T1D Exchange. RR is *Associate Editor for Journal of Diabetes*.
